# Potential of *Streptomyces avermitilis*: A Review on Avermectin Production and Its Biocidal Effect

**DOI:** 10.3390/metabo14070374

**Published:** 2024-06-30

**Authors:** Ernesto Cerna-Chávez, José Francisco Rodríguez-Rodríguez, Karen Berenice García-Conde, Yisa María Ochoa-Fuentes

**Affiliations:** 1 Departamento de Parasitología, Universidad Autónoma Agraria Antonio Narro, Calzada Antonio Narro 1923, Saltillo 25315, Coahuila, Mexico; ernesto.cerna@uaaan.edu.mx; 2 Estudiante de Postgrado en Ciencias en Parasitología Agrícola, Universidad Autónoma Agraria Antonia Narro, Calzada Antonio Narro 1923, Saltillo 25315, Coahuila, Mexico; d_karenb.garcia_c@uaaan.edu.mx

**Keywords:** antiparasitic, avermectin, biocide, macrocyclic lactones, *Streptomyces avermitilis*

## Abstract

Secondary metabolites produced by the fermentation of *Streptomyces avermitilis* bacterium are powerful antiparasitic agents used in animal health, agriculture and human infection treatments. Avermectin is a macrocyclic lactone with four structural components (A1, A2, B1, B2), each of them containing a major and a minor subcomponent, out of which avermectin B1a is the most effective parasitic control compound. Avermectin B1a produces two homologue avermectins (B1 and B2) that have been used in agriculture as pesticides and antiparasitic agents, since 1985. It has a great affinity with the Cl-channels of the glutamate receptor, allowing the constant flow of Cl- ions into the nerve cells, causing a phenomenon of hyperpolarization causing death by flaccid paralysis. The purpose of this work was to gather information on the production of avermectins and their biocidal effects, with special emphasis on their role in the control of pests and phytopathogenic diseases. The literature showed that *S. avermitilis* is an important producer of macrocyclic lactones with biocidal properties. In addition, avermectin contributes to the control of ectoparasites and endoparasites in human health care, veterinary medicine and agriculture. Importantly, avermectin is a compound that is harmless to the host (no side effects), non-target organisms and the environment.

## 1. Introduction

Microbial natural products, also known as secondary metabolites, are valuable compounds used in agriculture, as well as in the pharmaceutical, veterinary and food industries [1,2], which are produced by a variety of microorganisms such as bacteria and fungi [3]. *Streptomyces* species are Gram-positive, filamentous, spore-generating bacteria [family Streptomycetaceae, class Actinobacteria] [4] known to be prolific producers of a wide variety of biologically active secondary metabolites, likewise their importance lies in being one of the most studied genera, with important medical and agricultural applications [5,6,7]. These compounds express antibacterial, antifungal, antihypertensive, antiviral, antitumor, immunosuppressive and insecticidal action [8]. Streptomyces is characterized as an abundant source of pharmaceutical compounds including amino acids, sugars, fatty acids and terpenes, which utilize biochemical pathways to combine to form more complex structures through precise metabolic pathways [9]. The secondary metabolites generated by *Streptomyces* are synthesized by a group of enzymes encoded by the corresponding set of biosynthetic genes, which are transcriptionally restricted, although the physiological role of *Streptomyces* transcriptional regulators is not well defined [10,11]. The latter is because several species of this genus have the ability to control morphological differences as well as the production of secondary metabolites, so their biosynthetic genes are specifically regulated by related regulatory genes [12]. With great features, the biosynthesis of secondary metabolites generated by this bacterium is mediated by regulatory pathways that can be stimulated by vital, nutritional and environmental incentives for the cell [13]. About 800 *Streptomyces* species have been studied [14], 100,000 antibiotic compounds have been reported, of which 70–80% of the bioactive products are applied in the production of drugs, agrochemicals for pathogen control and plant development promoters [8,15,16]. The products obtained by *Streptomyces* are characterized by their structural diversity, such as aminoglycosides, ansamycins, glycopeptides, macrolides, terpenes and tetracyclines [8]. The species *Streptomyces avermitilis* stands out due to the diversity of whole genome sequencing studies, including the avermectin biosynthetic gene cluster [17], resulting in at least 8.7 million reference pairs. on the linear chromosome, as well as new information on the organization of avermectin biosynthetic genes [spanning a distance of 82 kb]. The increasing number of whole genome sequences of *Streptomyces* has revealed that we know only a fraction of the biosynthetic potential of this genus [6,18]. Bacteria often use small extracellular signaling molecules to control complex physiological functions, such as biofilm production, pathogenicity, and antibiotic production capacity [18,19]. Autoregulators are signaling molecules that can trigger antibiotic production in the genus *Streptomyces*. Genomic analysis of three genera of *Streptomyces*, *S. avermitilis*, *Streptomyces coelicolor* A3 [5,20] and *Streptomyces griseus* [21] has shown that these microorganisms have large linear chromosomes. which harbor more than 20 sets of secondary metabolic genes. These genes are involved in polyketide biosynthesis by polyketide synthases (PKS) and are required for peptide synthesis by non-ribosomal peptide synthases (NRPS), as well as for producing bacteriocins, terpenoids, shikimate metabolites, aminoglycosides, and other natural products. *S. avermitilis* is used on an industrial scale to produce avermectin, which has been shown to be a highly efficient secondary metabolite-based product and anthelmintic agent. Likewise, Ivermectin-dihydroavermectin B1 [22,23], has been used as an agricultural pesticide and antiparasitic agent since 1985, so the present research work aimed to gather information on the production of avermectins from *S. avermilitis* and its biocidal properties.

## 2. *Streptomyces avermitillis* Origin

In the early 1970s, the Kitasato Institute (now part of Kitasato University) in Japan, in cooperation with Merck & Co Inc. of the USA, developed a new class of antiparasitic agents [22]. Satoshi Ōmura, the Japanese parasitologist expert in isolating natural products, studied a group of soil-dwelling actinobacteria (*Streptomyces*), characterized by producing a large number of antibiotic, anticancer, antimicrobial, antiviral, antitumor, cytotoxic, herbicidal, immunosuppressive, insect control agents and plant growth promoters [3,23,24,25].

Ōmura isolated new *Streptomyces* strains from Japanese soil and cultured them in the laboratory, selecting fifty of the most active strains to test their therapeutic potential against pathogenic microbes. William Campbell, an expert in parasite biology, tested the efficacy of the compounds isolated by Ōmura. His results showed that *Streptomyces avermitilis* came from a golf course located in Ito, Japan and had outstanding efficacy against parasites of domestic animals [26]. Campbell conducted in vivo laboratory tests in which he found a compound with a novel, powerful and promising bioaction. This compound was named “avermectin” [1].

## 3. *Streptomyces avermitilis’* Main Secondary Metabolites

After the discovery of streptomycin by Selman A. Waksman, actinomycetes are considered the most fruitful source of new antibiotics; The most important classes of antibiotics for clinical use were developed between 1940 and 1960, from different soil microorganisms, nowadays, society is facing an emerging threat of microbial drug resistance, so the increased demand for new antibiotics of microbial origin has become a social and political problem [27].

The importance of the genus *Streptomyces* lies mainly in its ability to produce a wide range of secondary metabolites [28], these bioactive products are characterized by not being fundamental within the life cycle of the microorganism, however at the same time Provides an evolutionary benefit, due to its application as a weapon of control or chemical control against pathogens such as bacteria, fungi, viruses, insects, among others, through deterrence, inhibition and death, providing advantages such as adaptation depending on the habitat where it is found [23,29]. Among the species studied, *S. avermitilis* has been noted to be a highly efficient producer of secondary metabolites as anthelmiticidal agents, avermectins, a series of eight 16-membered pentacyclic lactones and oligomycins as major secondary metabolites [1,30,31]. 

Oligomycins are elaborate 26-membered macrocyclic lactones that produce strong toxic compounds that inhibit the oxidative phosphorylation reaction in mammalian cells [32]. Along with the production of these bioactives, *S. avermitilis* serves as a versatile host for heterologous production of secondary metabolites from other *Streptomyces* species, enhancing the yield and production of these bioactive compounds derived from more than 20 biosynthetic gene clusters (BGCs) [4,30,31]. 

The *S. avermitilis* genome has been sequenced and identified cryptic secondary metabolite pathways, which are not or weakly expressed under standard laboratory growth conditions [33,34,35,36], revealing a gap between their potential and observed biosynthetic gene expression. The gene clusters involved in the biosynthesis of *S. avermectillis* metabolites are generally contiguous, encoding enzymes responsible for the stepwise assembly of bioactive molecules. However, this group of silent or cryptic genes represents a potential source of new antimicrobial drug discovery [34,37]. Several techniques currently exist for the activation of silent genes in actinomycetes, such as in situ activation of these genes (promoter engineering, transcription factor operation and ribosome engineering), their expression in heterologous hosts (cloning, reconstruction of biosynthetic pathways and rational engineering of chassis stresses) [38,39,40,41], the systematic condition of culture parameters [42], co-culture [43] and the use of chemical elicitors, which induce antibiotic synthesis [44]. For their Tyurin et al. (2018) propose a new technique based on small organic molecules (γ-Butirolactones and their derivatives) at minimal concentrations (nanomolar to micromolar) to induce secondary metabolite biosynthesis in actinomycetes [45]. 

### 3.1. Macrocyclic Lactones

**Figure 1:**
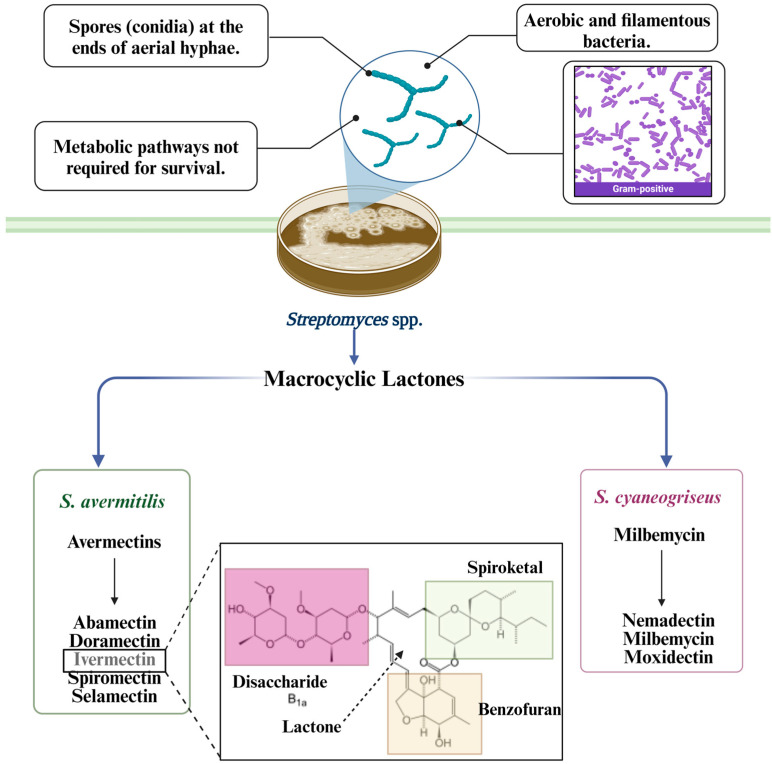
Important characteristics of *Streptomyces*. Production of macrocyclic lactones (avermectins and milbemycins) by *S. avermertilis* and *S. cyaneogriseus*, and chemical structure of ivermectin; [50,51,52,53]. Created with BioRender.com (accessed on 7 May 2024).

Interest in natural products, such as secondary metabolites produced by various microorganisms and plants, has been increasing, as they represent a wide range of compounds with inherent properties and specific and effective defense mechanisms against other organisms, being key in the development of bioactive substances [46,47]. Lactones are an important example of secondary metabolites due to their chemical composition and biological activity [48,49]. Lactones can be mainly classified into γ-Lactones, δ-Lactones, Medium-sized lactones, Phtalides, Coumarins, Spirolactones, Strigolactones, Macrolactones or Macrocyclic Lactones [49]. Macrocyclic lactones are cyclic esters that belong to two large families, depending on the original fermented actinomycetes: avermectins produced by *S. avermitilis*, and milbemycins produced by *S. cyaneogriseus* (Figure 1) [50,51]. The complex chemical structures of these drugs stem from a 16-membered macrocyclic lactone, similar to the macrocyclic lactone of macrolide antibiotics (but without the bacterial effect). Avermectins (abamectin, doramectin, eprinomectin, emamectin and ivermectin) share a 16-membered macrocyclic lactone backbone with different functional groups in the benzofuran, disaccharide and spiroketal moieties. Both families of macrocyclic lactones are highly lipid-soluble drugs.. Both families of macrocyclic lactones are highly lipid-soluble drugs. Macrocyclic lactones are large molecules with molecular weights ranging from 600 kDa (milbemycins) to 800 kDa (avermectins) [50]. In the last 35 years, these molecules have gained importance in the control of parasitic infections, but much remains to be learned about them.

**Figure 2:**
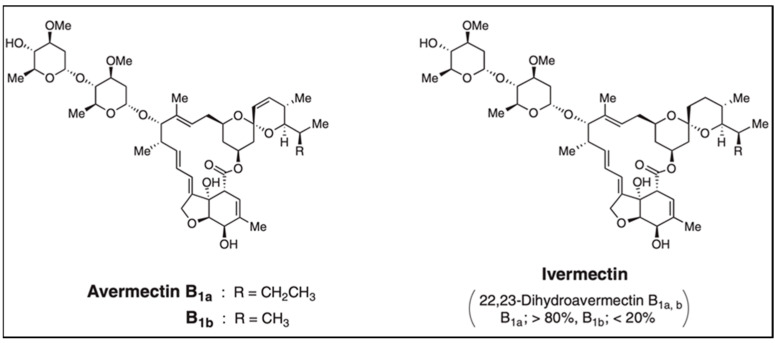
Chemical structure of avermectin and ivermectin [22].

Avermectins are divided into natural (Ivermectin and Abamectin) and biosynthetic (Doramectin, Eprinomectin and Selemectin) [54]. Milbemycins include milbemycin, moxidectin and nemadectin [55,56]. Ivermectin and Abamectin [Figure 2], were the first drugs used in the control of parasites [intestinal worms and arthropods] [57,58]. The first in vitro tests of ivermectin were performed at Merck Sharp & Dohme research laboratories with mice infected with the nematode Nematospiroides dubius, which indicated that the whole broth obtained from fermentation of the bacterium was highly effective in a range of at least eightfold without toxicity to rodents [1]. Subsequently, evaluations of the individual components were performed, and although there were differences in their effectiveness, component B1a proved to be active against other nematode species (*Trichostrongylus axei*, *Trichostrongylus colubriformis*, *Cooperia oncophora*, *Oesophagostomum columbianum*, *Haemonchus placei*, *Ostertagia ostertagi*, *T. axei*, *T. colubriformis*, *C. oncophora*, *Cooperia punctata*, *Oesophagostomum radiatum* and *Dictyocaulus viviparus*) with an oral dose of 0.1 mg kg^−1^ and in the case of canine hookworm (*Ancylostoma caninum*) with a dose of 0.005 mg kg^−1^ presented a control of 83 to 100% [59].

#### 3.1.1. Avermectin

Avermectin is a by-product of a pentacyclic compound with 16 members and a disaccharide made by Loleandrose units (1 → 4) linked to the macrolide ring in C13. Cane et al. (1983), suggested that avermectin aglycone has seven acetates, five propionates and one 2-methylbutyrate or isobutyrate and its biosynthesis follows the polyketide synthetases’ pathway (PKS) [60]. According to research, the anthelmintic activity comes from Avermectin produced by the mycelium of *S. avermitilis*. Chromatographic and spectrophotometric techniques were used to determine the four structural components of avermectin, [A1, A2, B1, B2] each with a major and a minor subcomponent [A1a, A1b; A2a, A2b; B1a, B1b; B2a, B2b]; resulting from the structural differences in C5, C22–C23 y C26 [61]. They are usually producing in ratios ranging between 80:20 and 90:10 [62]. Out of the eight main avermectin compounds, B1a is the most efficient compound against a broad range of nematodes and parasitic arthropods affecting domestic animals [63]. B1a forms two homologs, avermectin B1 and B2, differentiated by a methyl group that has been used as an agricultural pesticide and antiparasitic agent since 1985 [64], due to its low harmful effect on humans [2]. This compound forms odorless yellowish-white crystals [51,65], and has anthelmintic power similar to the power of Ivermectin or even higher [66]. It differs from Ivermectin only by the presence of a double bond in carbons 22 and 23 [51,65]. Since this compound showed activity against endoparasites and ectoparasites, it was called endectocide; a term currently applied to macrocyclic lactones in general. Merck & Co Inc. introduced this product for livestock use in Australia and it extended to other places as an agricultural pesticide due to its low cost [67]. 

Later, scientists working for Merck & Co Inc., developed a specific analog program for abamectin, seeking to identify an active compound that could work against a broad spectrum of *Lepidoptera*. As a result, they discovered emamectin, which was produced as benzoate salt (MK-244) [68], particularly effective against *Tuta absoluta* [69]. Emamectin comes from avermectin through a five-step synthesis process and it is far more powerful than avermectin [70]. Novartis S.A. de C.V. introduced emamectin benzoate to the market in 1997. In 2000 a 2007 emamectin benzoate was officially approved [71], as the only therapeutic chemical allowed for the control of parasites in salmon’s production [72], and at present it is also used in insecticides for agricultural pest control. Currently a new member of the avermectin family with a patent number (2012105478044), produced by Hebei Xingbai Agricultural Technology Co., Ltd., China, has been registered in China. This new compound is Abamectin B2 which is a mixture of B2a and B2b and is registered for the management of root-knot nematodes in crops such as tomato, cucumber, celery, watermelon, peanut, soybean, banana and coffee [73].

#### 3.1.2. Avermectin Biosyntesis

Due to the commercial importance of *S. avermitilis*, previous studies have characterized its genetic structure as well as the gene cluster that synthesizes Avermectin. The complete genome of *S. avermitilis* has at least 8.7 million base pairs on the linear chromosome [17]. The genes involved in avermectin synthesis are organized similarly to complex polyketides [74]. The nucleotide sequence has been determined with 18 ORF’s (Open Reading Frame) [75], encoding one cargo module and 12 extension modules at 82 Kb [76]. Four ORF’s (aveA1, aveA2, aveA3 and aveA4) encode for multifunctional polyketides, constituting the avermectin polyketide synthetase and the twelve enzymatic activity modules for polyketide chain elongation are generated, while aveC and aveE are related in polyketide modification and aveD and aveF encode a C5 O-methyltransferase and a C5-ketoreductase, respectively, which modify avermectin intermediates. For oleandrose synthesis, the aveBII and aveBVIII genes are related and the aveB1 gene is involved in macrolide biosynthesis [77]. 

**Figure 3:**
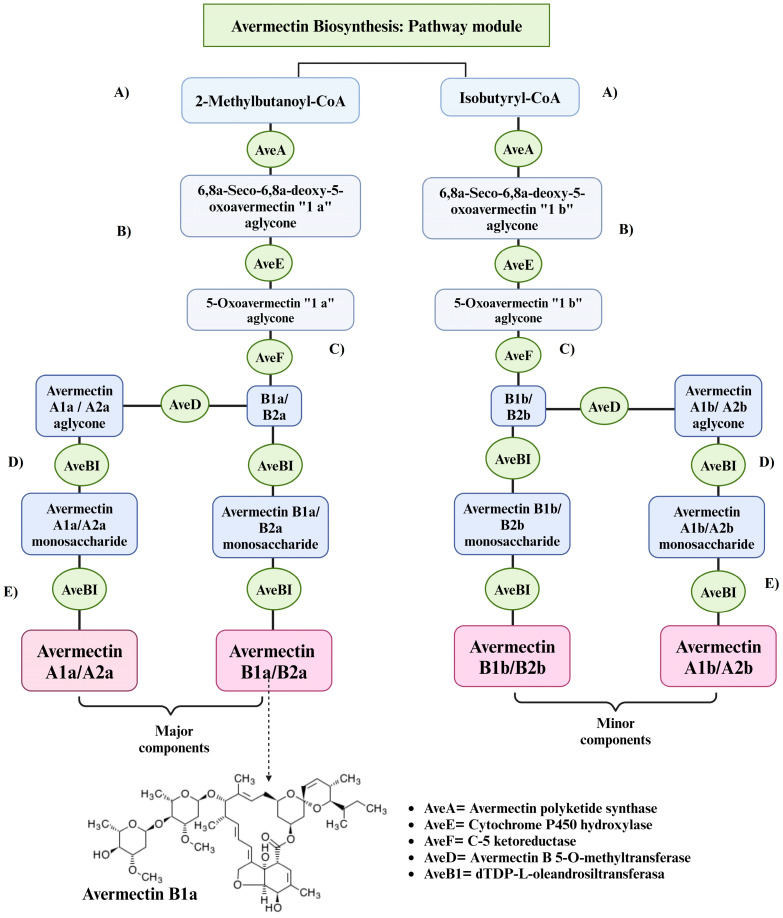
Avermectin biosynthesis; 2-methylbutanoyl-CoA => 6,8a-Seco-6,8a-deoxy-5-oxoarmectin 1a/1b aglycone => avermectin B1a. Avermectin biosynthesis consists of the following steps: (**A**) Elongation of a polyketide chain by four multifunctional modulating polyketide synthase components (AveA1,2,3,4); (**B**) Modification by dehydration of C22–23 and formation of spiroketal by AveC; (**C**) Furan formation and keto reduction by AveE and AveF15; (**D**) Biosynthesis of dTP-l-oleandrose by AveBI; and (**E**) Glycosylation of aglycones to form the final Avermectins (A1a, A2a, B1a, B2a, A1b, A2b, B1b and B2b) [53,79,80]. Created with BioRender.com (accessed on 7 May 2024).

The synthesis of avermectin proceeds in three steps: (1) formation of an aglycone, (2) modification of the aglycone to form aglycone avermectin and (3) glycosylation of the aglycone avermectin with a derivative of an oleandrose. An acyl group is derived from the catabolism of isoleucine in the “a” components and valinate from the “b” components [31], bind to 2-methylbutyryl-CoA or isobutyryl-CoA to convert the acetyl group and valinate to isobutyl or isopropyl for the “a” and “b” components, respectively [76], subsequently four additional peptides of polyketide synthetase (PKS) are responsible for the enzymatic activity to give rise to aglycone. The carbon chain undergoes several modifications, such as the formation of a furan ring and methylation to form the macrocyclic lactone and finally, a derivative of oleandrose one (oleandrose dioxythymidine diphosphate) is attached and thus generates avermectin [31]. Avermectin B1a (Figure 3), is the main component of avermectins, its application is largely directed to the control of internal anthelmintics, external parasites and for the control of agricultural pests, due to its broad spectrum of bioactivities [78].

Currently, the production of Avermectin is still a process exclusively by submerged fermentation (SmF) using different strains of *S. avermitilis* [81,82]. However, advances have been made that help to have a better control in the production of Avermectin. Cao and co-workers in 2018, established a high throughput screening (HTS) strategy integrated by fluorescence activated cell sorting and random mutagenesis to detect *S. avermitilis* mutant strains with high yields of Avermectin, such process reported advantages in efficient spore selection, reduction in labor process of HTS process and improvement in process accuracy [83]. In 2022, the increased production of Avermectin B1a using the high-yielding industrial strain of S. avermitilis A229 was studied using a combined strategy that provides an efficient approach by improving B1a production by 49.1% with the implementation of genetic engineering [84]. Tian and coworkers in 2024, investigated *MtrA* (*sav_5063*) gene which is a transcriptional regulator of the OmpR family in *S. avermitilis*, reporting a negative regulatory effect on Avermectin biosynthesis, indicating that it plays crucial functions in the coordination of physiological processes (growth, development and morphological differentiation) in *S. avermitilis*, being this an advance on the regulation of Avermectin biosynthesis [85]. 

#### 3.1.3. Avermectin Biocidal Propierties

The genus *Streptomyces* has the ability to produce a large number of secondary metabolites, including antibiotics and other biologically active compounds widely used in human health care, agriculture and veterinary medicine. Avermectin has been the most relevant of these compounds with biocidal action [86]. Initially, avermectin was considered effective against helminths, insects and spiders, without causing harm to flatworms, protozoa, bacteria and fungi [87,88]. However, recent studies have shown that it can also act against the genus *Mycobacterium* [89]. 

Avermectin has been used for more than 20 years to eradicate human diseases such as lymphatic filariasis [90]; onchocerciasis, one of the poorly treated tropical diseases in Africa [91,92] and strongyloidiasis in Asia [93]. In May 1977, Merck Co Inc. was asked to consider the potential use of avermectin in humans, given the demonstrated efficacy of ivermectin against uncinaria and other intestinal nematodes in dogs. In January 1978, data on filarial worms did not appear particularly promising due to the lack of effect on adult parasites [67]. Subsequently, avermectin proved to be a wonder drug for human health, improving the nutrition, general health and well-being of billions of people worldwide since it was first used against onchocerciasis in humans in 1988 [22]. It is the ideal drug in many ways. It is highly effective, safe, well tolerated; it is easy to apply and is currently used to treat various nematode-related internal infections, such as onchocerciasis, strongyloidiasis, ascaridiasis, filariasis, gnathostomiasis and trichuriasis. It is also part of oral treatments against ectoparasite infections, such as pediculosis [lice infestation] and scabies (mite) [94]. Currently, the pharmaceutical potential of avermectin includes treatments against *Mycobacterium tuberculosis*, such as multidrug-resistant tuberculosis and extensively drug-resistant tuberculosis [84]; as well as the synergistic effect of avermectin B1a with methicillin against methicillin-resistant *Staphylococcus aureus* [38,94]. Recent studies have shown that cytochrome P450 (CYP105D7) production by *S. avermectilis* can hydrolyze pharmaceutically important flavanones [naringenin and pinocembrin] [95], due to their antioxidant, anti-inflammatory and anticancer properties [96]. For example, we know that naringenin is a good inhibitor of aromatase (an important strategy in the treatment of breast cancer) [97,98]. On the other hand, naringenin in grapefruit juice has been shown to inhibit P450 metabolites that metabolize drugs in the human kidney [99].

In 1981, an injectable formulation of ivermectin was introduced in France for veterinary use as a subcutaneous treatment of cattle and a new injectable formulation was introduced in New Zealand for intramuscular treatment of horses (it was replaced in 1984 by oral formulations). Subsequent introductions included ivermectin for sheep in Brazil [1982] and for pigs in the UK (1983); abamectin for cattle in Australia (1985); and Ivermectin for dogs in the USA (1987) [67]. These drugs are safe, effective, low cost, easy to apply, with minimal side effects and show a broad spectrum of effectiveness against gastrointestinal nematodes, pulmonary nematodes and ectoparasites in domestic animals [100,101]. In addition, they have proven to be effective treatments for infections caused by worms, as well as mites, lice and scabies [102]. However, despite the benefits of these biopharmaceutical drugs, their broad spectrum of action has also raised concerns about their impact on non-target organisms in terrestrial and aquatic environments [103]. It is eliminated in the feces of treated animals [104], causing ecotoxicological effects on non-target organisms associated with the decomposition of organic matter such as beetles, flies, springtails, mites, earthworms and free-living nematodes [105], while Pérez-Cogollo et al. (2018) mentions that to reduce the amount of avermectin residues to the environment it is necessary to perform parasitosis diagnostics to apply selective treatments in bovine herds [106,107]. 

Within the applications in the agricultural area [Table 1], this type of compounds has been used in several countries for the control of agricultural pests [108], due to their powerful action as nematicides, acaricides and insecticides [109]. Today, it is used for chemical seed treatment for the control of plant parasitic nematodes [110], such as *Meloidogyne incognita* [111], *Pratylenchus zeae* [112], *Heterodera schachtii* [113], *Tylenchulus semipenetrans* [114], *Radopholus similis* [115], and *Bursaphelenchus xylophilus* [116]. They also exhibit a broad spectrum of action against pests. insects in socially important commercial crops, including mites and insects of the orders *Coleoptera*, *Hymenoptera*, *Diptera*, *Orthoptera*, *Isoptera* and *Lepidoptera* [117].

#### 3.1.4. Mode of Action

The biocides mentioned in this literature review have a non-systemic mode of action, but show good translaminar activity [127]. They act by ingestion and to a lesser extent by direct contact [128,129], although these biocides can be absorbed by all the usual routes, due to their high liposolubility. They are distributed throughout tissues, including the intestinal tract, fat and skin [130,131], acting as allosteric modulators of the glutamate-regulated chloride channel (GluCl) [132], by binding to a high affinity receptor, this binding increases the permeability of Cl-ions, causing a detachment of the parasite by flaccid paralysis. The identification of the specific receptor to which avermectin and emamectin benzoate bind has been controversial. Early studies claimed that the biocides produced a release of gamma-aminobutyric acid (GABA) from the synaptosomes of the rat brain; as well as modulation of GABA receptors that increased their affinity for the neural transmitter. Depending on the concentration of the toxicant to which the parasites are exposed, Cl- entry may or may not be mediated by the GABAergic mechanism [81,118,119,120,121,122,123,124,125,126].

**Figure 4:**
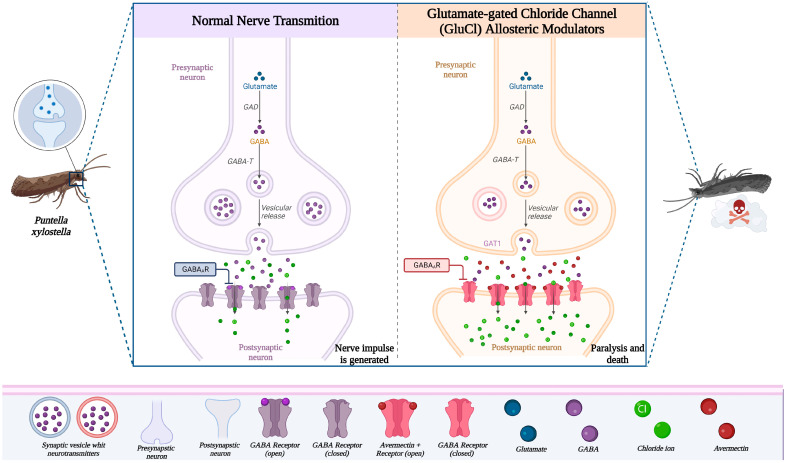
Normal nerve transmission and mode of action of Avermectins (allosteric modulators of the glutamate-regulated chloride channel (GluCl)) in *Puntella xylostella* [53,135]. Created with BioRender.com (accessed on 7 May 2024).

Recent research work suggests that the antiparasitic action of avermectins is due to their interaction with glutamate receptor-gated Cl- channels in the target parasite, giving rise to the phenomenon of hyperpolarization [51,133]. In fact, avermectin acts on the neural transmission of the parasite by binding to a glutamate receptor of chloride channels on neural cell membranes, close to a GABA receptor and a benzodiazepine receptor, minimizing GABA action; which increases GABA release and action potential [54]. The binding triggers the release of a flux of Cl- ions into the neural cells of the parasites that increases permeability, producing pharyngeal hyperpolarization and somatic muscle paralysis, leading to parasite death [51]. Olsvik et al. (2008) mention that toxicity in mammals is low, since avermectins do not cross the mammalian blood-brain barrier and therefore the GABA receptor does not affect the neurons of the central nervous system (Figure 4) [134].

## 4. Conclusions

*Streptomyces avermitilis* is a significant producer of macrocyclic lactones such as Avermectin, with substantial potential as a biocide in agricultural parasitology. The demonstrated efficacy of Avermectin against a wide range of agriculturally significant phytopathogens, along with its safety profile for crops and the environment, positions Avermectin as a promising tool in integrated pest management.

However, to maximize its use as a biocide, a deeper understanding of the mechanisms of action and pharmacokinetics in specific agricultural environments is necessary. This aims to better comprehend how such microbial-derived substances interact with phytopathogens and how they are distributed and degraded in the environment. Additionally, further research is required for the development of more efficient formulations and application strategies that optimize its effectiveness in pest and disease control, while minimizing any negative impact on non-target organisms (e.g., beneficial microorganisms) and the ecosystem as a whole.

With a continued focus on biotechnology and metabolic engineering, new opportunities can be explored to enhance the selectivity and efficiency of Avermectin as an agricultural biocide, paving the way for more sustainable and environmentally friendly agricultural practices.

## Figures and Tables

**Table 1:** Applications of macrocyclic lactones for the control of insects and nematodes of agricultural importance.

Compound	Application		Reference
Avermectin B2	Microcapsules	Population of RKN = Efficiency 80%	[118]
Ivermectin	Ivermectin 1% diluted in DMSO 5%	Susceptibility to EPN (*Steinernema* y *Heterorhabditis*)	[119]
Abamectin	Abamectin (18 g/L) diluted in N-Methyl-2-Pyrrolidone	Significant *Globodera pallida* control, soil application	[120]
Avermectin B1	Emamectin benzonate	Susceptibility of *Spodoptera fugiperda.*	[121]
Abamectin	Abamectin 1.8%, per 10 plants 2.5 mL	Efecto acaricida en ninfas de *Tetranicus* spp. en maíz	[122]
Avermectin	N,O-carboxymethylchitosan (NOCC) grafted whit avermectin	Insecticidal activity at 4 mg/L against *Spodoptera exigua*, *Tetranychus cinnabarinus* and *Aphis fabae.*	[123]
Avermectin B1a	40 avermectin derivates	Biological activity against *Tetranychus cinnabarinus*, *Aphis craccivora* and *Bursaphelenchus xylophilus*	[124]
Ivermectin B1a	25-methyl y 25-ethyl ivermectin	Nematicidal activity against *Caenorhabditis elegans*, and insecticidal activity against *Mythimna separata* larve.	[125]
Abamectin	Abamectin (95%) (avermectin B1a > 80% and avermectin B1b < 20%)	Time and dose dependet cell viability in *Spodoptera frugiperda.*	[126]
